# MTOR Promotes Astrocyte Activation and Participates in Neuropathic Pain through an Upregulation of RIP3

**DOI:** 10.1007/s11064-025-04341-x

**Published:** 2025-02-01

**Authors:** Bingru Dong, Danyang Li, Shasha Song, Na He, Shouwei Yue, Sen Yin

**Affiliations:** 1 Rehabilitation Center, Qilu Hospital of Shandong University, Jinan, 250000 Shandong China; 2 Institute of Rehabilitation Engineering, University of Health and Rehabilitation Sciences, Qingdao, 266000 Shandong China; 3 Department of Neurology, Qilu Hospital of Shandong University, Jinan, 250000 Shandong China

**Keywords:** Neuropathic pain, Astrocyte, Neuroinflammation, MTOR, RIP3

## Abstract

Neuropathic pain (NP), a chronic pain condition, is the result of abnormalities in both central and peripheral pain conduction pathways. Here, we investigated the underlying mechanisms associated with this effect. We found that following chronic constriction injury (CCI) surgery, there was an increase of mTOR in astrocytes and an activation of astrocytes within the spinal cord. Pharmacological inhibition of mTOR reversed CCI-induced hyperalgesia and neuroinflammation. Moreover, knockdown of astrocytic mTOR rescued the downregulation of spinal glutamate metabolism-related protein expression, underscoring the pivotal role of mTOR in modulating this pathway. Intriguingly, we observed that overexpression of mTOR, achieved via intrathecal administration of TSC2-shRNA, led to an upregulation of RIP3. Notably, pharmacological inhibition of RIP3, while ineffective in modulating mTOR activation, effectively eliminated the mTOR-induced astrocyte activation. Mechanistically, we found that mTOR controlled the expression of RIP3 in astrocytes through ITCH-mediated ubiquitination and an autophagy-dependent degradation. Taken together, our results reveal an unanticipated link between mTOR and RIP3 in promoting astrocyte activation, providing new avenues of investigation directed toward the management and treatment of NP.

**Supplementary Information:**

The online version contains supplementary material available at 10.1007/s11064-025-04341-x.

## Introduction

A prevalent, chronic pain condition that exerts a significant impact on the quality of life is that of neuropathic pain (NP). This condition results from disorders or injuries to somatosensory nerves [1]. While the underlying pathophysiology, which includes peripheral and central sensitization, is well understood, the fundamental mechanisms behind the development of NP remain unclear [2, 3]. Moreover, few effective treatments currently exist for the management of NP [2]. Accordingly, there exists an urgent need to identify the underlying mechanisms contributing to NP for the development of new and more effective treatments of this condition.

Central sensitization, a condition of synaptic plasticity and enhanced neuronal reactivity in central pain pathways following painful damage, plays a role in the maintenance of chronic pain [4]. There is mounting evidence that neuroinflammation in the central nervous system (CNS) drives central sensitization [5, 6]. The activation of glial cells in the brain and spinal cord, such as microglia and astrocytes, which results in the production of proinflammatory cytokines and chemokines, is a characteristic of neuroinflammation [6].

The most common type of cells within the CNS are astrocytes [7], and there is increasing evidence which suggests that astrocytes are involved in the development and maintenance of neuropathic or inflammatory pain [7–9]. In support of this suggestion are the findings that pain sensibility is reduced when astrocyte activity within the spinal cord is inhibited [10]. Astrocytes of the A1 subtype exert a neurotoxic effect through the release of pro-inflammatory mediators, whereas the secretion of anti-inflammatory mediators by the A2 subtype has a neuroprotective effect [11, 12]. However, the intracellular and intercellular signaling pathways involved in this astrocyte activation are not yet understood.

One such target for consideration is that of mammalian target of rapamycin (mTOR), which plays a role in biological processes such as protein translation and gene transcription [13]. Notably, hyperactivation of astrocytic mTOR in the tuberous sclerosis complex increases the release of gliotransmitters and thus promotes neuronal hyperexcitability [14]. However, whether mTOR is involved in the astrocyte activation associated with NP remains unknown. Other notable findings as related to the present study include the observation that an inhibition of mTOR alleviates demyelination and neuroinflammation in globoid cell leukodystrophy [15]. In addition, receptor-interacting protein 3 (RIP3), which is involved in the development and responses to tissue injury, antiviral immunity and many other physiological and pathologic processes [16]. RIP3 is expressed at a high level in reactive astrocytes after spinal cord injury [17], and neuroinflammatory response is induced by the activation of RIP3 after perceived cellular stress [18]. On the contrary, inhibition of RIP3 of the spinal cord could reduce neuropathic pain [18]. Since both mTOR and RIP3 are associated with inflammation, their relationship has attracted much attention but not in NP. As there is evidence indicating that mTOR can upregulate RIP3, as demonstrated in enterocolitis [19] and tuberous sclerosis complex [20], we hypothesized that mTOR may induce astrocyte activation by regulating the expression of RIP3.

In this study, we focused on mTOR’s function in A1/A2 astrocyte transformation and central sensitization in the spinal cord of a rat model of chronic constriction injury (CCI) of the sciatic nerve. In addition, we further assessed the potential for mTOR to regulate astrocyte activation through an upregulation of RIP3.

## Materials and Methods

### Animals

Adult male Wistar rats weighing 150–180 g were obtained from the Laboratory Animal Center at Shandong University. All the rats were housed 3–4 per cage, had free access to food and water and were maintained under conditions with a temperature of 22 ± 2 °C and a 12-h light/dark cycle. The animals were acclimatized to these conditions for 7 days prior to use in the experiment. Strict procedures were implemented to mitigate the pain and suffering and the least number of rats needed to perform valid statistical analyses were used in each part of the study.

### Materials

See Supplementary Table 1.

### CCI Surgery

After anesthetizing the rats with sodium pentobarbital (50 mg/kg body, intraperitoneal injection), the lateral portion of the sciatic nerve was exposed by bluntly dissecting the left biceps femoris muscle. At the proximal end of the trigeminal branch of the sciatic nerve, approximately 6 mm of the nerve was separated from the adherent tissue. Four surgical wires were then used to ligate the nerve at intervals of approximately 1 mm, resulting in a ligation extending 4.5 mm in length along the nerve. An identical procedure was performed in rats of the Sham group, with the exception that the sciatic nerve was not ligated. After surgery, the muscle, lumbar dorsal fascia and skin were sutured sequentially. Surgeries were performed under aseptic conditions. Rats demonstrating self-mutilation or total sensory loss following the surgery were eliminated from the experiment.

### Behavioral Tests

Animals were subjected to behavioral testing on their ipsilateral hind paw both before and after surgery (Supplementary Figure S1). For the behavioral tests, rats were placed in individual Plexiglas containers with a wire mesh at the bottom and acclimatized to this test environment for approximately 30 min as described previously [21]. With use of a mechanical pain meter (kw-CT-1, Calvin biotechnology, Nanjing, China), sufficient force was applied vertically to the middle plantar surface of the left hind foot with von Frey filaments. The demonstration of a rapid retraction or licking of the foot were considered as positive responses when assessing the paw withdrawal threshold (PWT).

To assess their paw withdrawal latency (PWL), rats were positioned inside a 5-mm thick Plexiglas container. Following a 30-min acclimatization period, radiant heat was applied through the glass to the surface of the left hindfoot using a thermal nociceptor (KW600 Calvin Biotechnology, Nanjing, China). In accordance with the method used by Hargreaves et al. to evaluate thermal nociceptive sensitization [22], the PWL was defined as the time between the application of radiant heat and the onset of a positive response as indicated by a licking or retracting of the hind paw. An automated 20-s timer was set to terminate the radiant heat if the rat did not withdraw its rear paw in order to avoid tissue damage. The experiment was replicated three times with 5-min intervals between each test. The mean score obtained in the three tests was used for statistical analyses. To reduce experimental bias, behavioral tests were conducted under blind conditions.

### Intrathecal Injection

Intrathecal injections into the subarachnoid space between the L4 and L5 vertebrae were administered to sedated rats using a 10-gauge needle. The precise site was confirmed by the observation of tail flicking. The mTOR inhibitor, rapamycin (RAPA, 50 μg/kg), and the RIP3 inhibitor, GSK872 (GSK, 100 μg/kg), were dissolved in DMSO and administered once daily on days 7–9 after CCI surgery.

The siRNA against rat TSC2 (target sequence: GTGCTGGAAGCTGATGCGAAA) and the triple-target tandem siRNA against rat mTOR (target sequence: AGGAGTCTACTCGCTTCTATG-ATTGAGTTGGGCTCTCTCACTTCT-CCAAGTGGAACTGCTTATCA) were synthesized by the Shanghai Genechem Technology Co.. Lentiviral vectors LV-GFP-TSC2-RNAi and LV-GFAP-EGFP-MIR155(mTOR)*3–1 containing the GFAP-specific promoter were constructed and stored at − 80 °C. Following removal from the freezer the virus solutions were dissolved by gentle shaking at 37 °C and the titer of the virus solution was adjusted to 1 × 10^8^ TU/mL with saline. Rats in each group were injected intrathecally at one week prior to CCI surgery. The negative control (NC) virus and lentiviral vector groups were injected intrathecally with 10 μl volumes for 10–30 s of NC-shRNA and lentiviral solution, respectively. The needle remained in place for 5 min following the infusion and was then slowly retracted. The L4–L5 spinal dorsal horn and the dorsal root ganglion (DRG) were collected at 3 weeks after virus inoculation to allow for a maximal and stable expression of their effects.

### Cell Culture and Treatments

Human embryonic kidney 293 T cells (CRL-3216, ATCC) and the rat astrocyte cell line (CTX TNA2, Jennio Biotech) were cultivated in DMEM supplemented with 10% endotoxin-free fetal bovine serum and incubated at 37 °C in 95% O_2_ and 5% CO_2_. Cells were grown to 70–80% fusion prior to treatment.

For the in vitro experiments, astrocytes were incubated with GSK (5 μM) for 24 h, chloroquine (CQ, 10 μM) for 18 h, SAR405 (10 μM) for 24 h or MG132 (10 μM) for 6 h, with Earle’s balanced salt solution (EBSS) medium applied for 0, 6 or 12 h to induce autophagy via amino acid starvation.

### Transfection

Astrocytes were grown to 20% fusion before transfection with LV-TSC2-shRNA. Lentiviral suspensions (moi = 5) were diluted with complete medium and the infection enhancement solution HiTransG P as provided by the reagent vendor was added. The cells were then incubated at 37 °C and after 16 h the complete medium was replaced and the incubation continued. GFP fluorescence in cells was monitored by fluorescent microscopy (Leica, Solms, Germany) at 72 h after transfection. Puromycin (2 μg/ml) was used to screen the cells, which were then subcultured.

ITCH-siRNA and NC-siRNA were transfected into astrocytes. The positive strand sequence of ITCH-siRNA was 5′-GAGCAAUGCAGCAGUUUAATT-3′ and the antisense strand sequence was 3′-UUAAACUGCUGCAUUGCUCTT-5′. Furthermore, pcDNA3.1-RIP3-C-FLAG and pcDNA3.1-ITCH-C-HA were transfected into 293 T cells. The lipofectamine 3000 reagent was used according to the manufacturers’ instructions. Cells were cultivated to 70–80% fusion prior to transfection. After fully combining the siRNA or plasmids with lipofectamine 3000, it remained at room temperature for 20 min at which time DMEM was added to the cells. The medium was changed to the complete medium 24 h after transfection, before any treatment was administered.

### Co-Immunoprecipitation (Co-IP)

Cell lysates were prepared using IP buffer (200:1 mixture of IP Cell lysis buffer and Phenylmethanesulfonyl fluoride). RIP3 (1 μg) and IgG (1 μl) antibodies were added to the IP samples and the mixture was incubated for 2 h on a rotator at 4 °C. Each sample received 40 μL of protein A/G agarose beads, which were rotated overnight at 4 °C. Samples were eluted three times with IP Cell lysis buffer. IgG was utilized as the negative control.

### Western Blotting

The L4–L5 spinal dorsal horn tissue samples and cultured cells were homogenized in RIPA lysis buffer. Proteins were obtained by centrifugation at 12,000 rpm for 20 min at 4 °C and were then applied onto a polyvinylidene fluoride membrane after being separated on a 10% SDS-PAGE gel. The following antibodies were used to incubate the membranes overnight at 4 °C after they were blocked with 5% skim milk: Rabbit anti-mTOR, Rabbit anti-p-mTOR, Rabbit anti-GFAP, Rabbit anti-RIP3, Rabbit anti-GAPDH, Rabbit anti-GS, Rabbit anti-TSC2, Mouse anti-Ub, Rabbit anti-ITCH, Rabbit anti-p62, Rabbit anti-FLAG, and Rabbit anti-HA antibody. The blots were visualized with use of an enhanced chemiluminescence system (Millipore) and Image J software (National Institutes of Health, MD, USA) was used to analyze the signal intensities.

### Real-Time Quantitative Polymerase Chain Reaction (RT-qPCR)

RT-qPCR was used to assess gene expressions in the tissue samples or cells. With use of RNA extraction kits, total RNA was extracted from these tissue samples or cells (Fastagen Biotech, Shanghai) and cDNA was then synthesized in accordance with the guidelines provided by the manufacturer (GeneCopoeia). The pre-denaturation step was performed at 95 °C for 30 s. Then, 40 cycles of 95 °C for 10 s and 60 °C for 30 s each comprised the PCR amplifications. Target gene expression levels in each sample were normalized using GAPDH mRNA expression, and relative mRNA levels were assessed using the comparative CT method (2-ΔΔCT). Supplementary Table 2 lists the primer sequences that were employed (Biosune, China).

### Immunohistochemistry Analysis

The L4–L5 spinal cord tissue samples were preserved in 4% paraformaldehyde, and then embedded and sectioned into tissue slices with a thickness of 5 μm. Slices were rehydrated in graded alcohol solutions after being deparaffinized in xylene. The primary antibody: Rabbit anti-TNF-α antibody, was incubated with these spinal cord slices. After incubation with a secondary antibody at 37 °C for 60 min, the sections were treated in diaminobenzidine (DAB, ZSGB-BIO technology, Beijing) for 60 min and then counterstained with hematoxylin. The immunohistochemistry images were obtained with use of a biological microscope (Nikon, Tokyo, Japan) and ImageJ software was used to calculate optical densities to quantify these results. The absolute intensities, as obtained with the control group, were used to standardize each group’s measurements with the Image J software.

### Immunofluorescence Staining

For immunofluorescence, the spinal dorsal horn, the DRG or cells were incubated with primary antibodies overnight at 4℃: Rabbit anti-p-GFP, or Rabbit anti-TSC2, or Rabbit anti-mTOR, or Rabbit anti-p-mTOR, or Rabbit anti-p-JNK, or Goat anti-C3d, or Rabbit anti-c-fos, or Rabbit anti-RIP3, or Rabbit anti-p62, and Mouse anti-p-mTOR, or Mouse anti-GFAP, or Mouse anti-IBA1, or Mouse anti-NEUN, or Mouse anti-ITCH antibody. Following three PBS washes, the sections or cells were incubated for 1 h at room temperature with a combination of fluorescence-conjugated secondary antibodies (1:200, Abbkine) and were then were stained with DAPI for 5 min. Images were captures with use of a Nikon fluorescent microscope. ImageJ software was used to quantify results of sections as described previously [23]. The total number of double-positive cells within a 436.18 μm*327.14 μm section of the spinal dorsal horn was counted and the number of double-positive cells per square millimeter was calculated. For double fluorescence staining of cells, Image J software was used to provide quantitative values for the analyses of fluorescent intensities. Absolute intensities were standardized, as based on the absolute intensity of the control group.

### Statistical Analysis

GraphPad Prism software, version 6 (GraphPad Software, San Diego, CA, USA) was used to analyze all the data, which were expressed as the means ± standard errors. An unpaired Student’s t-test was used for comparisons involving two groups while a one-way analysis of variance (ANOVA) was used for comparisons involving three or more groups, with Bonferroni’s test used for post-hoc pairwise comparisons following a statistically significant ANOVA. For all analyses, a P < 0.05 was required for results to be considered as statistically significant. All analyses were performed by an investigator who was blinded as to the source of the data.

## Results

### mTOR in Astrocytes Mediates CCI-Induced NP

**Fig. 1:**
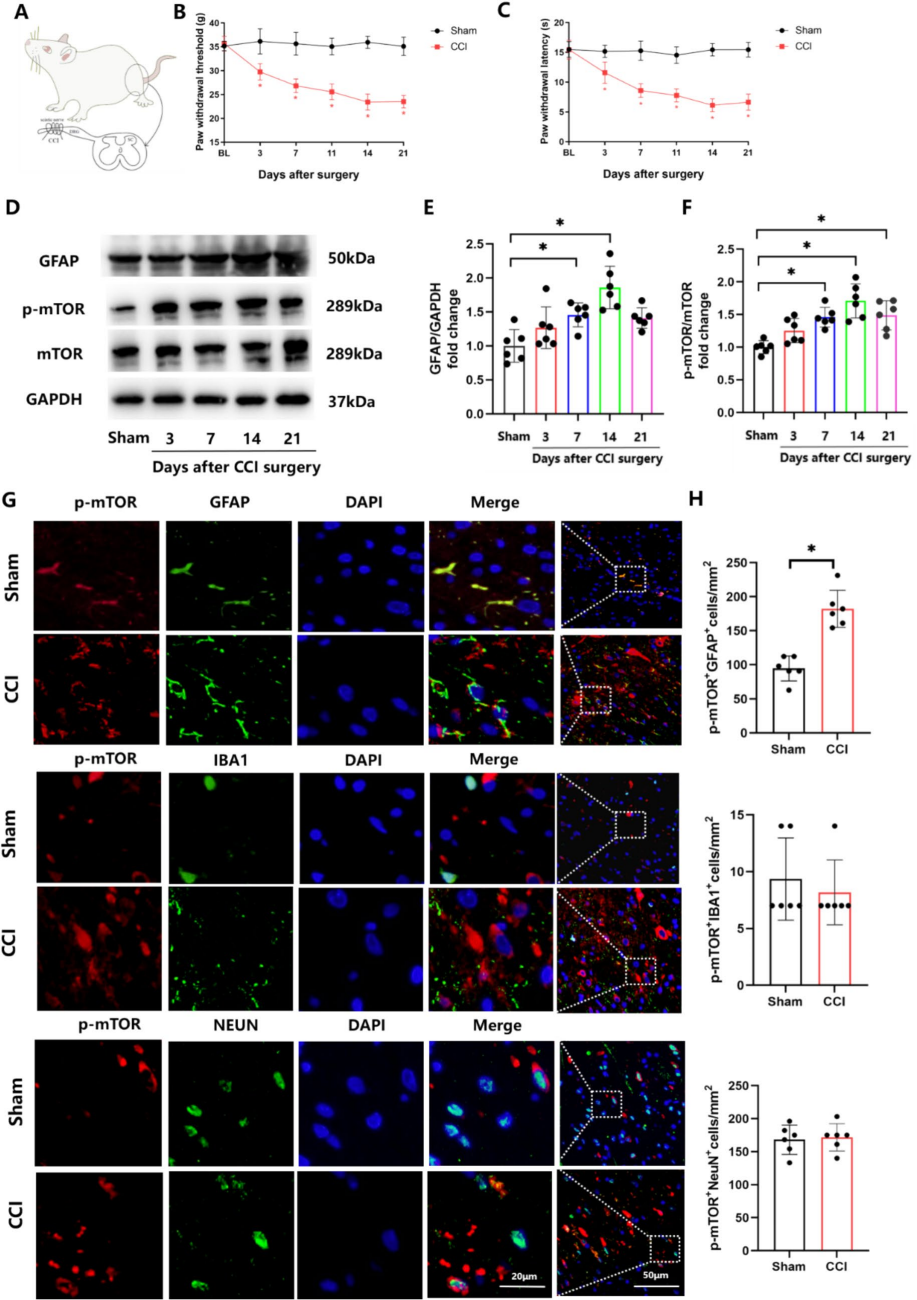
p-mTOR is upregulated and astrocytes are activated in a CCI-induced rat model of NP. **A** Protocol for generating the CCI-induced rat model of NP. **B**, **C** Mechanical allodynia and thermal hyperalgesia in the ipsilateral hind paw were determined by calculating the paw withdrawal threshold (PWT) and latency (PWL) on days 0, 3, 7, 11, 14 and 21 post-CCI. **D**–**F** Western blotting and quantification for the ratios of p-mTOR to mTOR and GFAP to GAPDH in sham and CCI rats. F(E) = 14.67, F(F) = 15.26. **G**, **H** Double-immunofluorescence of p- mTOR (red staining) and GFAP, IBA1 and NEUN (green staining) in the spinal cord of sham and CCI rats. Histogram illustrated that p- mTOR co-localization with GFAP was increased in CCI rats. (*p < 0.05, n = 6 in each group)

To investigate the role of mTOR in NP, we carried out CCI surgery (Fig. 1A). There were no statistically significant differences in PWT and PWL between the sham and CCI groups prior to surgery, however, significant decreases in PWT and PWL were obtained in the CCI versus control group from d3 to d21, with maximal differences being observed on d14 (Fig. 1B, C). These results suggest that the CCI surgery successfully induced NP. Results from our WB showed that protein levels of p-mTOR and glial fibrillary acidic protein (GFAP) were elevated in L4–L5 spinal cord tissue samples of rats in the CCI group. Additionally, p-mTOR protein expression in the rat spinal cord following CCI surgery was consistent with GFAP (Fig. 1D–F). To identify the types of cells expressing mTOR in the dorsal horn of the spinal cord, we co-stained p-mTOR with neuronal (NEUN), microglial (IBA1) and astrocyte (GFAP) markers. Immunofluorescent assay results revealed that p-mTOR was predominantly expressed in neurons and astrocytes and this expression of p-mTOR was elevated in astrocytes, but not neurons, after CCI (Fig. 1G–H). These data suggest that mTOR is specifically activated in astrocytes, but not neurons, within the L4–L5 spinal cord region after CCI.

### Pharmacological Inhibition of mTOR Markedly Alleviated CCI-Induced Allodynia and Neuroinflammation

**Fig. 2:**
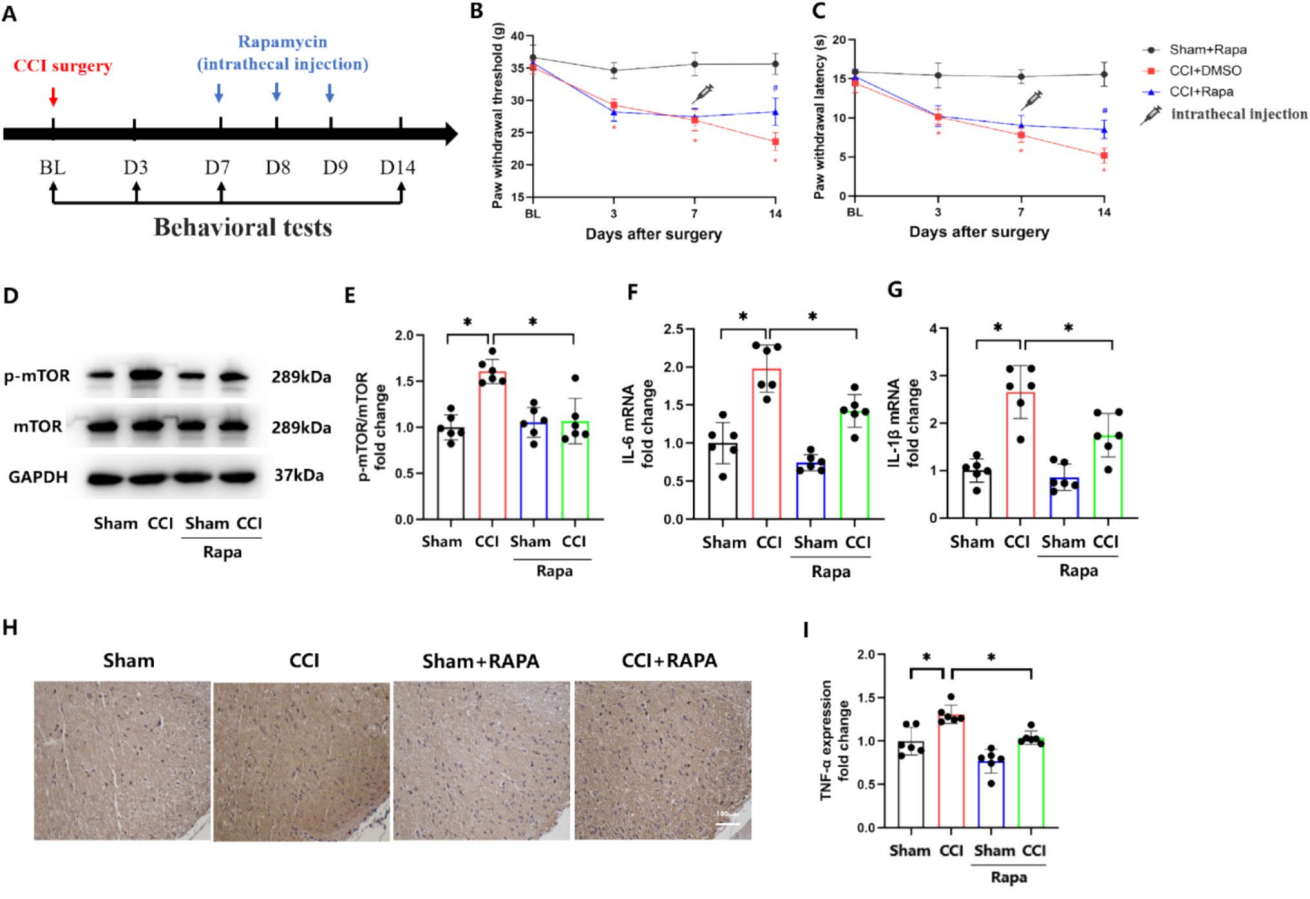
Inhibition of MTOR prevents CCI-induced allodynia and neuroinflammation. **A** An illustration of the experimental scheme. **B**, **C** Mechanical allodynia and thermal hyperalgesia in the ipsilateral hind paw was determined using the PWT and PWL on days 0, 3, 7 and 14 post-CCI in rats treated with rapamycin. **D**, **E** Western blotting and quantification for the ratios of p-mTOR to mTOR in sham and CCI rats treated with the mTOR inhibitor, rapamycin. F = 15.95. **F**, **G** mRNA levels of IL-6 (F) and IL-1β (G) in CCI rats treated with rapamycin. F(F) = 30.98, F(G) = 24.72. **H**, **I** TNF-α was assessed using immunohistochemistry in sham and CCI rats treated with rapamycin. F = 19.22. (*p < 0.05, n = 6 in each group)

To further characterize the role of mTOR in NP, RARA was intrathecally injected into CCI rats (Fig. 2A). Not surprisingly, RAPA markedly alleviated mechanical allodynia and thermal hyperalgesia (Fig. 2B, C) and downregulated CCI-elevated p-mTOR (Fig. 2D, E). In addition, the increased expressions of inflammatory factors interleukin 6 (IL-6) (Fig. 2F), interleukin 1β (IL-1β) (Fig. 2G), and tumor necrosis factor-α (TNF-α) (Fig. 2H, I), as neuroinflammation biomarkers in NP [24, 25], were also tested by RT-qPCR and immunohistochemical in CCI rats. However, the CCI-induced inflammatory response was relieved by RAPA. These findings imply that neuroinflammation in NP may be related to mTOR.

### Knockdown of Astrocytic mTOR Rescues the Impairments of Spinal Glutamate Clearance and the Increases in Activity of Dorsal Horn Neurons Resulting from CCI

**Fig. 3:**
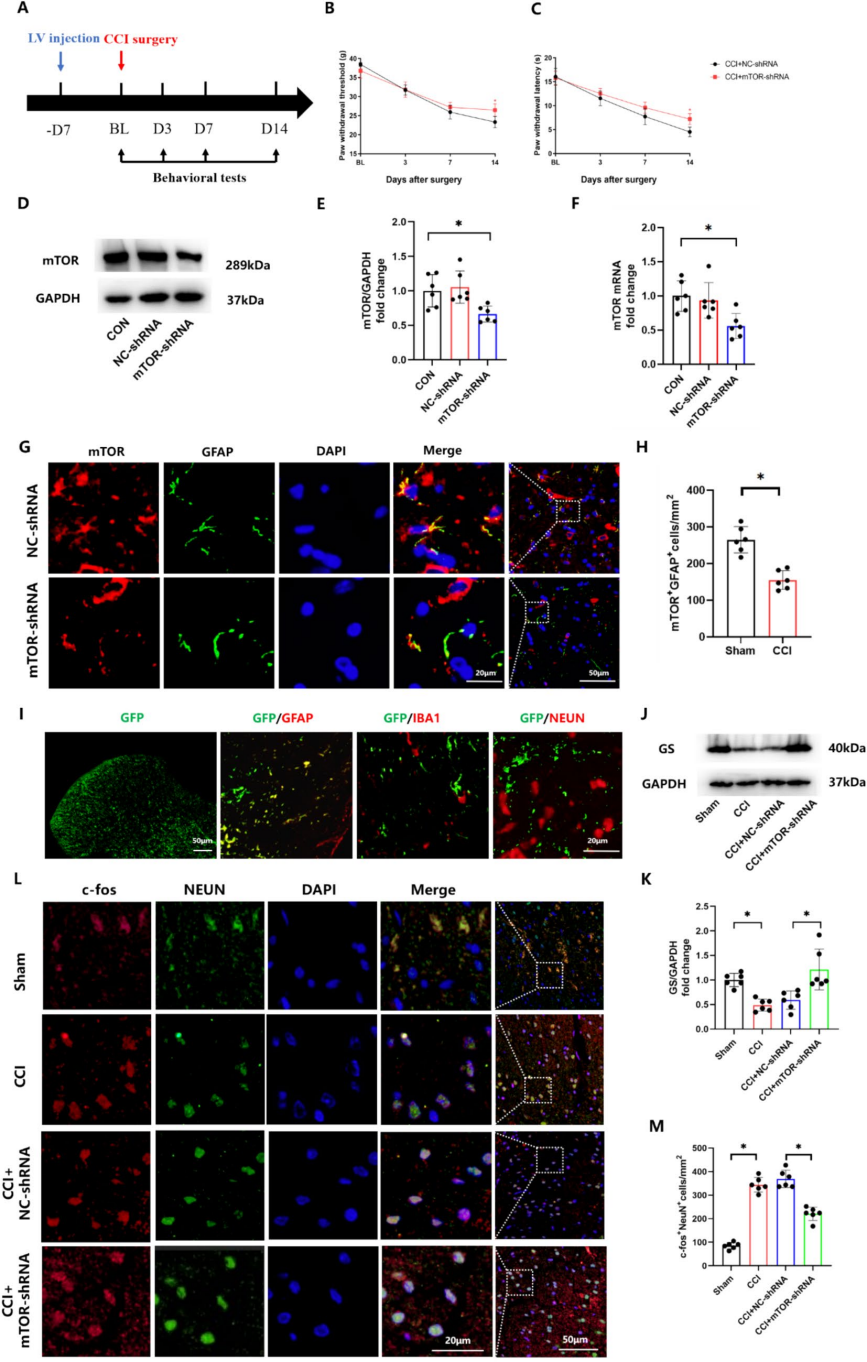
Astrocytic reduction of mTOR increases glutamate clearance and attenuates spinal dorsal horn neuron activity induced by CCI. **A** An illustration of the experimental scheme. **B**, **C** Effect of a reduction in the expression of astrocytic mTOR on mechanical allodynia and thermal hyperalgesia as evaluated using PWT and PWL. **D**, **E** Western blotting and quantification of the mTOR to GAPDH ratio in the spinal dorsal horn of rats with a reduction in the expression of astrocytic mTOR. F = 11.57. **F** mRNA levels of the mTOR in the spinal dorsal horn of rats with a reduction in the expression of astrocytic mTOR. F = 6.698. **G**, **H** The double-immunofluorescence of mTOR (green staining) with GFAP (red staining) in the spinal dorsal horn. Histogram illustrated that mTOR co-localization with GFAP was decreased in rats treated with mTOR-shRNA. **I** The colocalization immunofluorescence of GFP (green staining) with GFAP (red staining), IBA1 (red staining), and NEUN (red staining). **J**, **K** Spinal glutamine synthetase (GS) expressions were determined and quantified using Western blotting. F = 11.68. **L**, **M** Double-immunofluorescence of c-fos (red staining) and NEUN (green staining) in each group. c-fos–positive neurons were quantified and normalized to that of the vehicle group. F = 121.3. (*p < 0.05, n = 6 in each group)

To determine the molecular basis of NP induction after activation of astrocytes, we injected LV-GFAP-eGFP-mTOR-shRNA intrathecally into rats at one week prior to CCI as an approach to specifically silence mTOR expression in spinal astrocytes (Fig. 3A). Behavioral responses of these rats were tested until 14 days after CCI surgery. As expected, an intrathecal injection of mTOR-shRNA significantly reduced CCI-induced NP (Fig. 3B, C). After completion of behavioral testing, L4–L5 spinal cord and DRG samples were harvested on the 21st day after LV injection. Results from our WB, PCR, and immunofluorescent assays within the spinal dorsal horn showed that mTOR expression levels were downregulated as compared with that observed in the NC group (Fig. 3D–H), and GFP was specific specifically expressed in astrocytes (Fig. 3I). In addition, GFP in the DRG was mainly co-localized with the satellite cell marker, GFAP, after intrathecal injection (Supplementary Figure S1). Taken together, these results indicate that the expression level of mTOR in spinal astrocytes had been successfully downregulated.

With regard to spinal cord glutamate clearance, we found that glutamine synthetase (GS), a marker of glutamate clearance, was reduced after CCI, while silencing of mTOR partially rescued GS expression (Fig. 3J, K). In addition, our results demonstrating that c-fos-positive neurons were increased by CCI, but significantly reduced by the knockdown of astrocytic mTOR, demonstrate that mTOR exerts a major influence upon the activity of spinal cord dorsal horn neurons (Fig. 3L, M). Taken together, these results suggest that reduced spinal glutamate clearance and increased activity of dorsal horn neurons after CCI are closely associated with a mTOR-induced activation of astrocytes.

### Pharmacological Inhibition of RIP3 Blocks the mTOR Overexpression-Induced Activation of Astrocytes

**Fig. 4:**
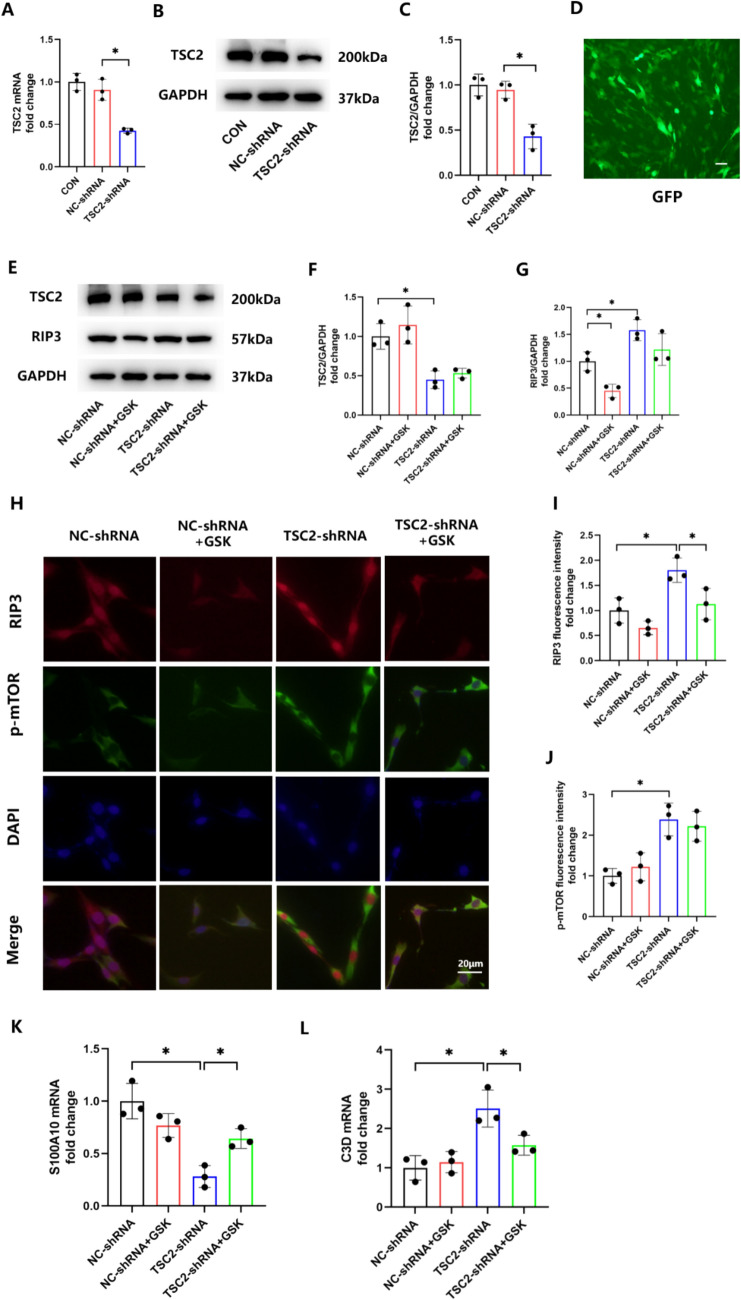
Pharmacological inhibition of RIP3 blocks the mTOR overexpression-induced astrocyte activation. **A** mRNA levels of TSC2 in astrocytes before and after transfection with TSC2-shRNA. F = 32.76. **B**, **C** Protein expression of TSC2 in astrocytes before and after transfection with TSC2-shRNA. F = 21.21. **D** GFP fluorescence in astrocytes transfected with TSC2-shRNA. **E**–**G** Western blotting and quantification of ratios of TSC2 and RIP3 to GAPDH in astrocytes treated with TSC2-shRNA or add GSK872 (5 μM) for 24 h. F(F) = 13.89, F(G) = 15.51. **H**–**J** Immunofluorescent staining of p-mTOR (J) and RIP3 (I) in astrocytes treated with TSC2-shRNA or add GSK872 (5 μM) for 24 h. F(I) = 11.68, F(J) = 12.97. **K**, **L** mRNA levels of S100A10 (K) and C3d (L) in astrocytes treated with TSC2-shRNA or add GSK872 (5 μM) for 24 h. F(K) = 17.61, F(L) = 12.15. (*p < 0.05, n = 3 in each group)

Based on these results, we sought to investigate the mechanisms by which mTOR acts. RIP3, which accumulates in reactive astrocytes in response to spinal cord injury [17], has also been shown to be associated with mTOR in enterocolitis [19]. Based on these findings, we hypothesized that the neuroinflammation, as induced by an overexpression of mTOR in astrocytes, results from an upregulation of RIP3. To this end, as the TSC1/TSC2 protein complex represents a major negative upstream regulator of mTOR [26], we transfected astrocytes with TSC2-shRNA to increase mTOR expression and employed RT-qPCR, WB and fluorescent labeling to verify the effect of this viral transfection (Fig. 4A–D). As shown in Fig. 4E–G, RIP3 was upregulated in the TSC2-shRNA group. Results from our immunofluorescent assay substantiated the effects, and showed us that mTOR activation was not affected after treating cells with the RIP3 inhibitor, GSK872 (Fig. 4H–J). In addition, the decreased S100A10 expression and the increased C3d expression, as demonstrated with RT-qPCR, established that mTOR overexpression induced the activation of A1 astrocytes, an effect that was reversed by GSK872 (Fig. 4K, L). These data suggest that RIP3 plays an important role in mTOR-induced astrocyte activation.

### RIP3 is Required for mTOR-Induced Neurotoxic Astrocyte Activation

**Fig. 5:**
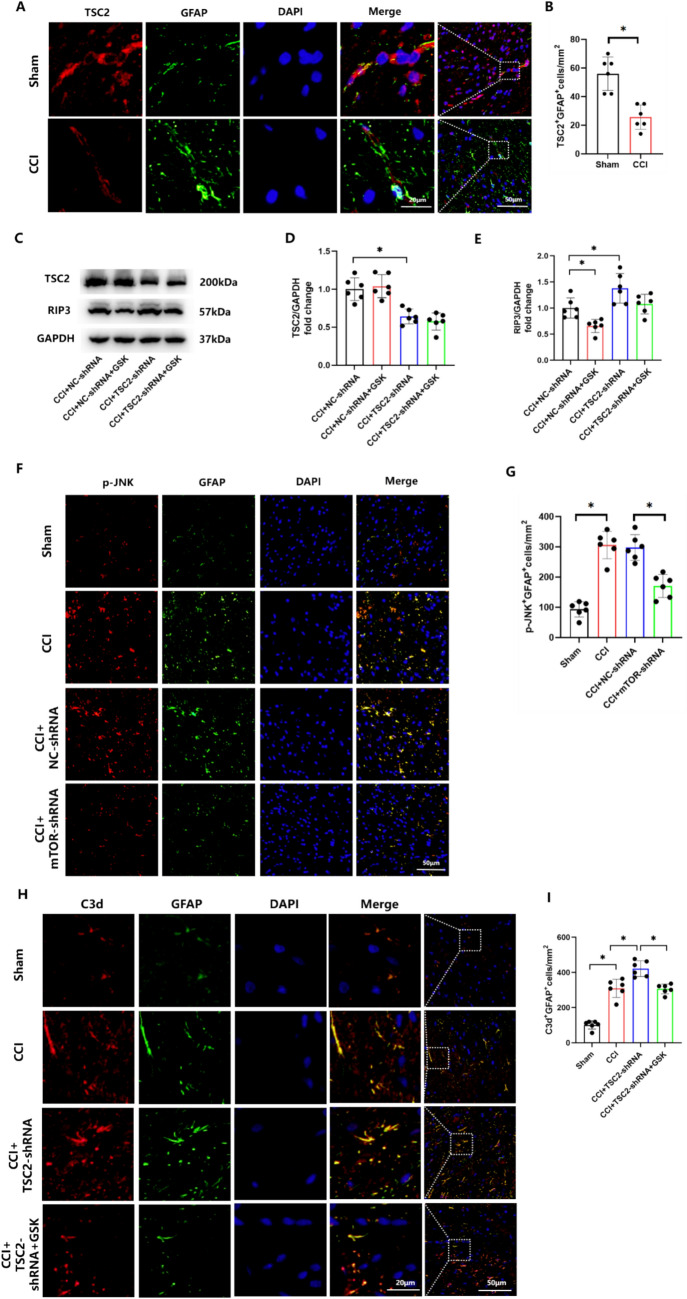
RIP3 is required for mTOR-induced neuroinflammation and astrocyte activation. **A**, **B** Double-immunofluorescence of TSC2 (red staining) and GFAP (green staining) in the spinal dorsal horn of sham and CCI rats. **C**–**E** Western blotting and quantification of the ratios of TSC2 and RIP3 to GAPDH in CCI rats treated with TSC2-shRNA or add GSK872. F(D) = 20.34, F(E) = 12.47. **F**, **G** Double-immunofluorescence showed the expression of p-JNK (red staining) and GFAP (green staining) in sham and CCI rats treated with NC-shRNA or mTOR-shRNA. F = 43.03. **H**, **I** Double-immunofluorescence images showing colocalization of GFAP-labeled astrocytes (green staining) and C3d-labeled A1 astrocytes (red staining) in CCI rats treated with TSC2-shRNA alone or with GSK872. F = 71.40. (*p < 0.05, n = 6 in each group)

To further investigate the role of RIP3 in NP induced by mTOR overexpression, we first examined the levels of RIP3 in rats of the CCI group. As demonstrated with WB, RIP3 expression levels were increased in the CCI group (Supplementary Figure S2A, B) and downregulated by RAPA (Supplementary Figure S2C, D). We also determined TSC2 levels within the sham and CCI group. Our immunofluorescent assay results revealed that the expression of TSC2 was reduced in astrocytes within the spinal dorsal horn in CCI rats (Fig. 5A, B). Subsequently, we injected TSC2-shRNA intrathecally into CCI rats and found that after silencing TSC2 to overexpress mTOR, RIP3 was upregulated in the spinal dorsal horn (Fig. 5C–E). Further, immunofluorescent assay results showed that an overexpression of mTOR promoted RIP3 activation in dorsal horn astrocytes of CCI-treated rats (Supplementary Figure S3A, B).

Previous reports have shown that nerve injury or spinal cord injury can induce activation of JNK in spinal astrocytes [27]. RIP3 participates in CCI induced activation of spinal astrocytes and neuropathic pain through phosphorylation of JNK [18]. Therefore, we further investigated the effect of mTOR on p-JNK using immunofluorescent staining. Our results showed that mTOR-shRNA injection inhibited JNK activation in spinal astrocytes of CCI rats (Fig. 5F, G). In addition, as C3d is a marker for the activation of neurotoxic responsive astrocytes (A1s) [28], we employed immunofluorescence to assess C3d/GFAP co-localization. We found that this co-localization, which was significantly increased in the CCI group, was further increased in the CCI + TSC2-shRNA group as compared with the sham group. However, GSK872 reduced the increased C3d/GFAP co-localization (Fig. 5H, I). Together, these results demonstrate that mTOR activation induces astrocyte activation in CCI-induced NP by upregulating RIP3.

### ITCH is Involved in the mTOR Regulation of RIP3 Ubiquitinated Degradation

**Fig. 6:**
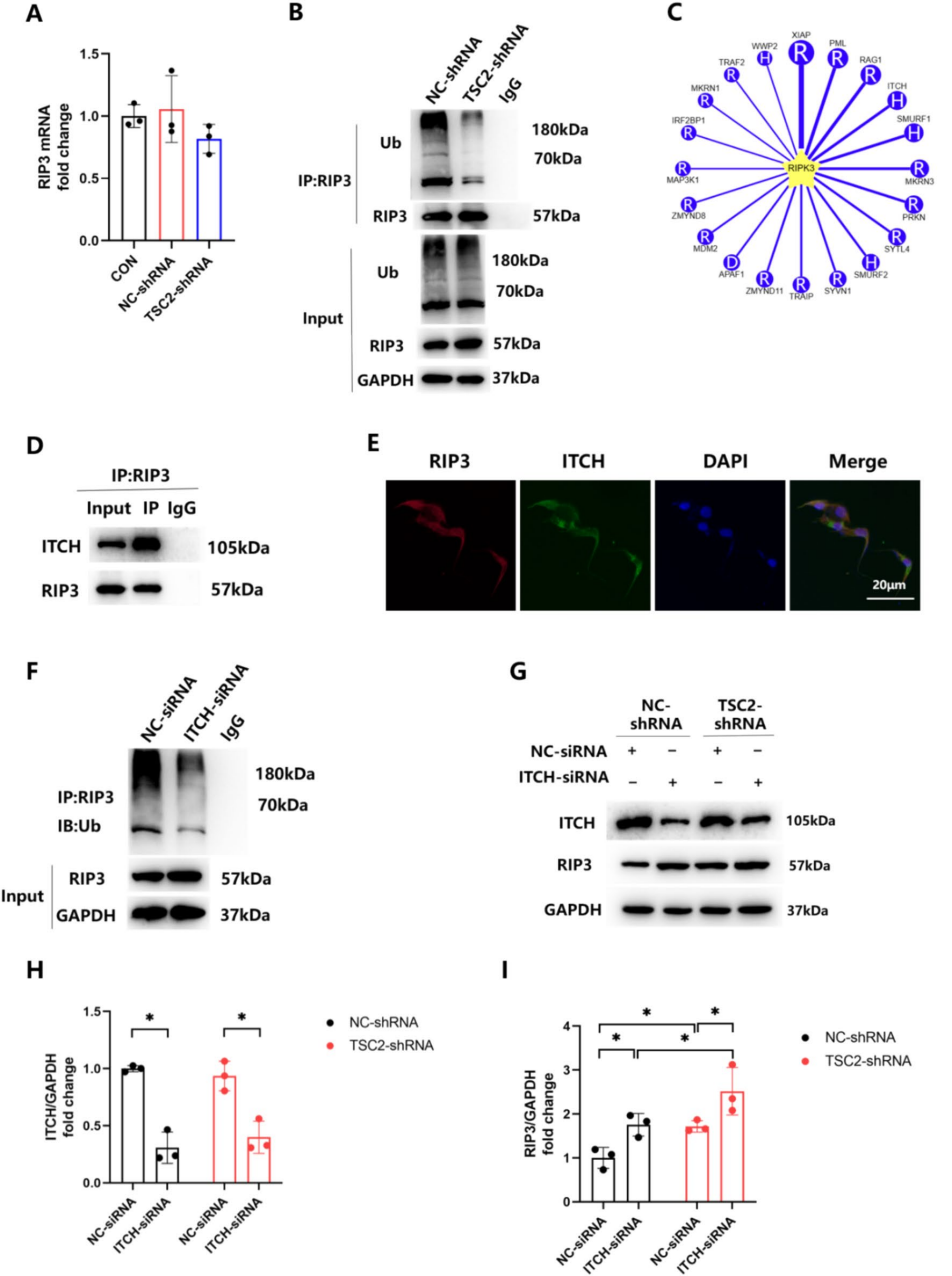
MTOR modulates the ubiquitinated degradation of RIP3 via ITCH. **A** mRNA levels of RIP3 in astrocytes before and after transfection with TSC2-shRNA. F = 1.501. **B** Cell lysates from astrocytes transfected with NC-shRNA or TSC2-shRNA were immunoprecipitated with normal IgG or anti-RIP3 antibodies, then immunoblotted with their respective antibodies. **C** The top 20 E3 ligases of RIP3. **D** Prepare cell lysates and immunoprecipitate with normal IgG or anti-RIP3 antibodies. Co-IP showed the interaction of ITCH with RIP3. **E** Endogenous RIP3 (red staining) colocalized with ITCH (green staining) in WT astrocytes. **F** Lysates of cells treated with NC-siRNA or ITCH-siRNA were subjected to immunoprecipitation with normal IgG or anti-RIP3 antibodies. **G**–**I** Western blotting demonstrated the ITCH dependency of mTOR mediated RIP3 ubiquitination. (*p < 0.05, n = 3 in each group)

Considering the critical role of RIP3 activation in this pathway, we continued to investigate how mTOR affects RIP3 levels. Transfection of astrocytes with TSC2-shRNA resulted in an overexpression of RIP3 (Fig. 4E, G), but did not upregulate RIP3 mRNA levels (Fig. 6A). In addition, we observed a significant increase in RIP3 ubiquitination within WT cells, but a significant decrease in TSC2-shRNA cells (Fig. 6B), thus suggesting that mTOR may regulate this process through E3 ubiquitin ligases. The UbiBrowser database was then used to predict E3 ligases for RIP3 [29, 30], and the top 20 E3 ligases are shown in Fig. 6C. Although we failed to show any interaction between XIAP/PML/RAG1 and RIP3 with use of Co-IP (Supplementary Figure S4A-C), we did find that ITCH was enriched when RIP3 was targeted by IP (Fig. 6D), results which clearly demonstrate an interaction between ITCH and RIP3. Moreover, endogenous RIP3 and ITCH were also found to be co-localized in the cytoplasm of astrocytes (Fig. 6E). As a means to further evaluate the regulatory role of ITCH on RIP3, astrocytes were transfected with ITCH-siRNA to knockdown ITCH (Supplementary Figure S4D, E) and we also examined whether ITCH could function as an E3 ligase for RIP3. Ubiquitination of RIP3 was reduced upon ITCH silencing (Fig. 6F), indicating that ITCH can act as an E3 ligase to regulate RIP3 ubiquitination degradation. As expected, the knockdown of ITCH promoted RIP3 upregulation as induced by mTOR overexpression, whereas an overexpression of mTOR interfered with ITCH-mediated RIP3 degradation, suggesting an inhibitory role of mTOR in ITCH function (Fig. 6G–I).

### The mTOR/ITCH Axis Regulates RIP3 Ubiquitination Degradation via the Autophagy Pathway

**Fig. 7:**
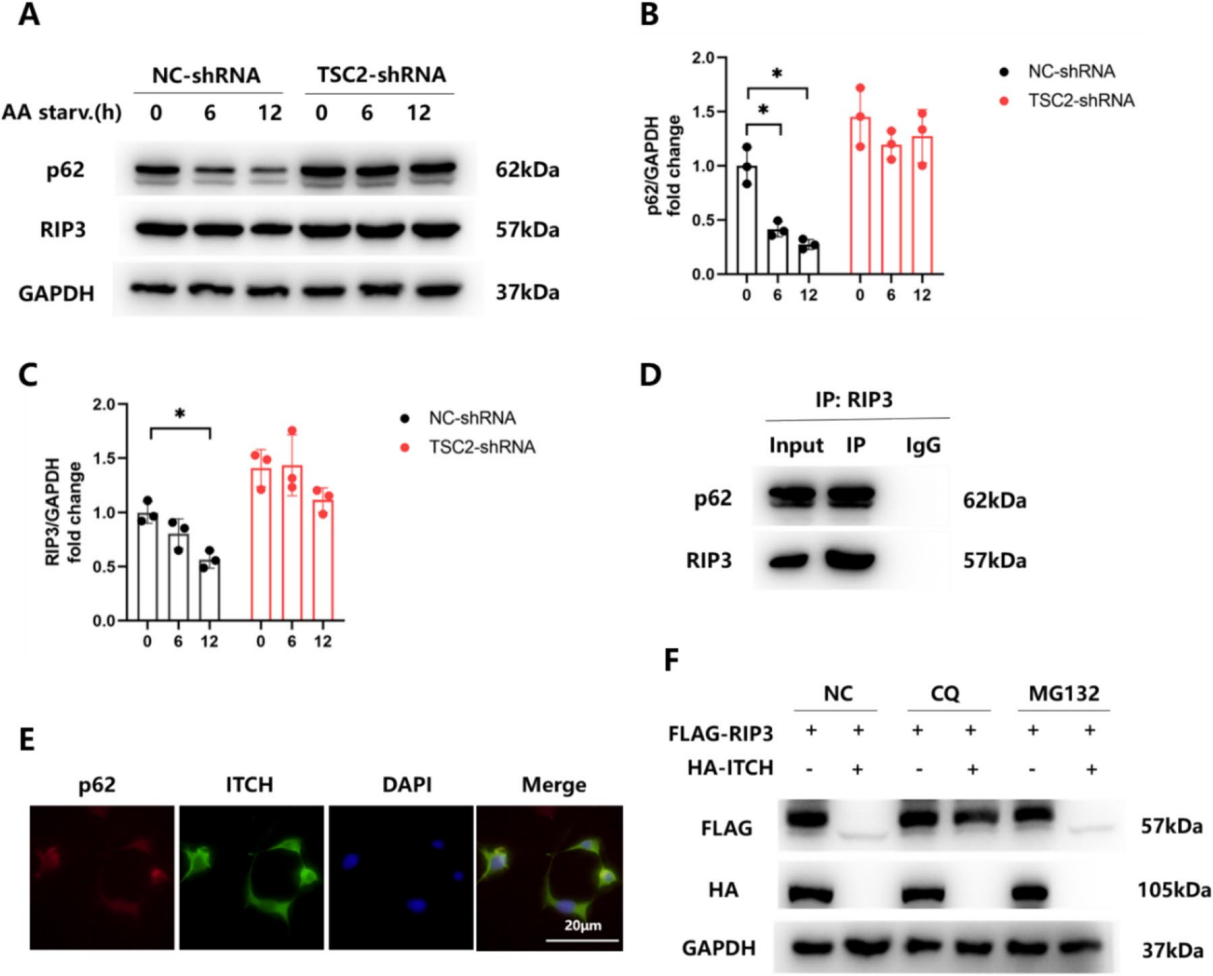
The mTOR/ITCH axis modulates the ubiquitination and degradation of RIP3 via the autophagy pathway. **A**–**C** RIP3 and p62 protein levels were determined using western blotting of cell lysates from NC-shRNA and TSC2-shRNA astrocytes treated with amino acid–free Earle’s balanced salt solution (EBSS) medium at the various times indicated. **D** Prepare cell lysates and immunoprecipitate with normal IgG or anti-RIP3 antibodies. **E** Endogenous p62 (red staining) colocalized with ITCH (green staining) in WT astrocytes. **F** HEK 293 T cells overexpressing FLAG-RIP3 alone or with HA-ITCH were either treated or not with 10 μM chloroquine (CQ, 18 h) or 10 μM (MG132, 6 h), respectively, with cell lysates then probed with anti-RIP3. (*p < 0.05, n = 3 in each group)

Normally, protein degradation relies on two degradation pathways, the ubiquitin–proteasome system (UPS) and the autophagy lysosome [31]. In this series of experiments our goal was to determine which pathway mediates the ITCH-induced ubiquitination degradation of RIP3. In WT astrocytes, the autophagy inducer, EBSS (amino acid starvation), produced a rapid degradation of p62, thereby establishing that autophagy was present (Fig. 7A, B). While these WT cells showed RIP3 degradation after amino acid starvation, no RIP3 degradation was observed in cells lacking TSC2, suggesting that autophagy triggered by amino acid starvation was ineffective in these latter cells (Fig. 7A, C). These data demonstrate that mTOR can exert an inhibitory effect on autophagy in TSC2-shRNA cells. As demonstrated with use of CO-IP and immunofluorescence, p62 and RIP3 can form a complex (Fig. 7D, E), which suggests that p62 plays a role in identifying ubiquitylated cargo proteins that are targeted for autophagic destruction when they are co-localize with astrocytes. When 293 T cells were transfected with FLAG-RIP3 and HA-ITCH plasmids, followed by treatment with CQ (autophagosome blocker) and MG132 (proteasome blocker), we found that ITCH-mediated RIP3 degradation was disrupted by CQ, but not MG132 (Fig. 7F), suggesting that ITCH specifically regulated RIP3 degradation via autophagy. Next, we co-cultured NC-siRNA and ITCH-siRNA cells with EBSS and the Vps34 inhibitor, SAR405, and then examined the levels of p62 and RIP3. While both cells showed comparable amounts of autophagy, the ITCH-siRNA cells showed significantly less RIP3 degradation in response to an identical exposure of amino acid starvation (Supplementary Figure S5A-C). Moreover, SAR405 was successful in blocking the degradation of RIP3 and p62 (Supplementary Figure S5A-C). Taken together, these findings imply that the mTOR/ITCH axis regulates ubiquitination degradation of RIP3 through the autophagy pathway.

## Discussion

**Fig. 8:**
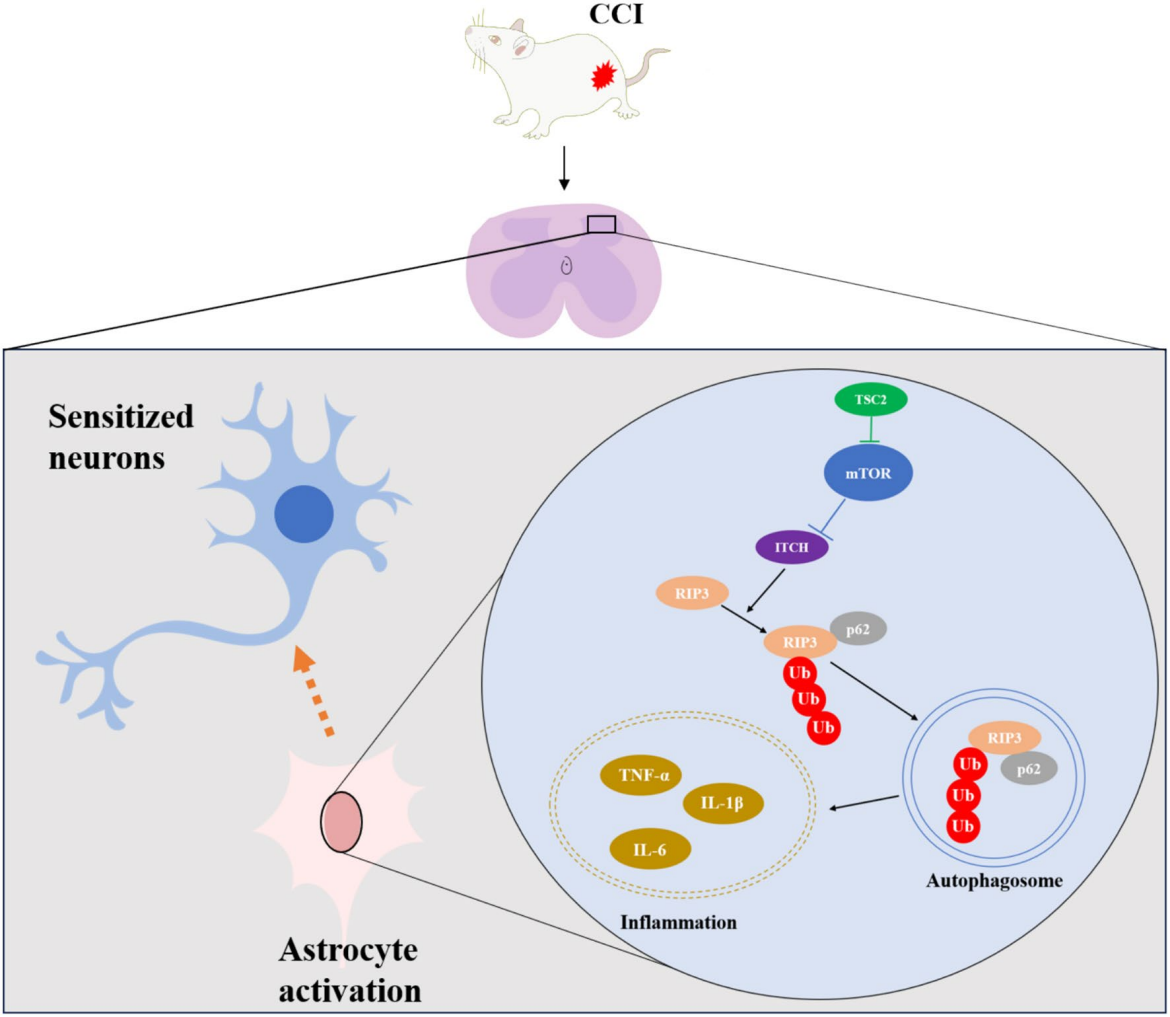
A functional model demonstrating how neuropathic pain is induced by astrocytic mTOR. Note: mTOR is overactived following the loss of TSC2’s negative regulation of it after chronic constriction injury, which results in the inhibition of ITCH activity and autophagy biogenesis. Due to the lack of degradation, RIP3 can be highly accumulated in the astrocytes, leading to the release of inflammatory factors. Finally, neuroinflammation induced the increased neuronal activity and enhancement of neuropathic pain

In the present study, we identified a previously unreported mechanism by which mTOR activation is involved in CCI-triggered NP. Specifically, we show that mTOR activation inhibits the ITCH-driven ubiquitinated degradation of RIP3, induces reactive activation and polarization of spinal astrocytes toward the A1 subtype, triggers neuroinflammation and generates central sensitization (summarized in Fig. 8). These findings provide robust evidence that mTOR-driven pain may involve an astrocytic-neuronal communication pathway, thus revealing the potential for the development of novel therapeutic targets.

The underlying mechanisms of NP are quite complex and involve both peripheral and central sensitization, making it refractory and unmanageable with current treatments [1]. It is well known that the mTOR signaling pathway is responsible for regulating transcription, translation and ribosomal biosynthesis [13]. There are reports showing that mTOR signaling in the spinal cord is required for neuronal plasticity and behavioral hypersensitivity in the rat after spinal nerve ligation (SNL) surgery [32]. Blocking mTOR improves NP evoked by spinal cord injury (SCI) [33]. Central NP is partially induced by the TRPM7/mTOR signaling pathway in astrocytes after SCI [34]. However, the role of mTOR in the development of neuropathic pain after CCI is still not fully understood. In the present study, we observed that during CCI-mediated NP development, mTOR activation was mainly observed in spinal astrocytes. Reactive astrocytes are known to produce a number of pro-inflammatory mediators, such as TNF-α, IL-6, neurotrophic factors, and neurotoxic substances like nitric oxide (NO) [9]. mTOR kinase regulates several intracellular processes in astrocytes, such as the rate of iNOS mRNA degradation [35] and the mRNA degradation of NO synthase in a degradable form [36]. Thus, mTOR is required for astrocyte activation and their role in neuroinflammation and NP. Moreover, a pharmacological inhibition of spinal mTOR or a specific knockdown of mTOR in astrocytes were both found to be effective in significantly alleviating nociceptive hypersensitivity. Such findings imply that astrocytic mTOR substantially contributes to CCI-induced NP.

In addition to enhancing central sensitization, activation of astrocytes also contributes to CNS neuroinflammation, which in turn leads to the development and maintenance of NP [37]. Reactive astrocytes have been reported to be categorized into two phenotypes, C3d A1 and S100A10 A2, which exert neurotoxic and neuroprotective effects, respectively [11, 12]. Numerous signaling pathways are involved in the transformation of astrocytes from their normal state to the A1 phenotype. According to Li et al., microglial cells can produce a transformation of astrocytes to the A1 phenotype in chronic postoperative pain via controlling CXCR7/PI3K/Akt [38]. Autocrine effects may also be important in astrocyte activation, as evidenced by the fact that astrocyte activation persists significantly longer than the peak of microglial activation and, in fact, can still occur in the absence of microglia [8, 39]. In our current study we found that the proportion of C3d-positive astrocytes was elevated in the spinal cord of rats after CCI surgery and that an overexpression of mTOR, using TSC2-shRNA, further promoted the activation of type A1 astrocytes. These findings provide compelling evidence indicating a critical role for mTOR in the activation of type A1 astrocytes.

The neurotoxic capacity of A1 astrocytes has been widely discussed, but little is known about their potential function in NP [36]. It has been reported that activation of the NF-κB signaling pathway in astrocytes during CNS inflammation generates NO which, when accumulated in excess, can exert a negative impact on neurons [40]. Moreover, under conditions of chronic pain, activated astrocytes become less capable of absorbing the excessive amount of glutamate released from neurons and other astrocytes. Under normal conditions, the glutamate transporter proteins, GLT-1 and GS, are primarily responsible for mediating this uptake [35]. However, the excitotoxicity of neurons, resulting from a prevention of glutamate uptake, can induce NP [41]. In a mouse model of bone cancer pain the expression level of spinal GLT-1 steadily declined as the disease progressed [42]. Our experimental results are consistent with this finding as we observed that the expression of GS proteins in the spinal cord was significantly reduced after CCI. A reduction in these proteins may be directly responsible for the reduced pain threshold and central sensitization observed in these rats. Interestingly, a decrease of mTOR in astrocytes inhibited glutamate release within the spinal cord, which is consistent with the previously reported role of mTOR in regulating glutamate metabolism after the onset of status epilepticus [43, 44]. In addition, pain research frequently uses c-fos, a marker of neuronal activity after injurious stimuli that is mostly expressed in the nucleus of injurious sensory neurons [45]. Our results strongly suggest that knockdown of astrocytic mTOR downregulates c-fos-positive neurons.

An additional novel and significant finding resulting from this study is the revelation that the astrocytic involvement of mTOR in NP occurs via an induction of RIP3. As demonstrated in a number of studies, RIP3 is implicated in the production and maintenance of NP and inflammation and thus may represent a viable target for pain management [18, 46]. Intrathecal injection of RIP3 inhibitors alleviated mechanical and thermal pain behavior induced by Complete Freund’s Adjuvant [47]. Paclitaxel promotes macrophage infiltration, leading to the release of TNF-α and IL-1β in DRG, and causing neuropathic pain through RIP3 [48]. Additional evidence indicating a relationship between RIP3 and astrocytes has been provided by Fan H et al. who reported that RIP3 accumulated and persisted in reactive astrocytes for up to 2 weeks after spinal cord injury [17]. We found that a clear link appears to exist among RIP3, reactive astrocytes and NP when collating these findings while organizing these studies, but few studies have been directed toward investigating this relationship. Here, we show that RIP3 was upregulated in rats subjected to CCI surgery, while an inhibition of mTOR, as achieved with RAPA, decreased RIP3 expression. Our findings that GSK872 mitigated mechanical and thermal nociceptive hypersensitivity after CCI and that neuroinflammatory responses were also greatly enhanced are supported by the study by Liang YX et al. [49]. We also found that GSK872 functioned without affecting TSC2 expression, implying that mTOR is an upstream regulator of RIP3. Overall, it seems reasonable to conclude that mTOR can induce astrocyte activation and produce inflammatory factors by increasing RIP3 expression.

More specifically, our findings imply that mTOR regulates RIP3 mainly at the post-transcriptional level, despite the fact that both transcriptional and post-transcriptional pathways can control RIP3 expression. In this study, with use of the UbiBrowser database [29], ITCH was identified as a candidate E3 ubiquitin ligase for RIP3. Although ITCH has been reported as an E3 ubiquitin ligase that recognizes a wide range of substrates and functions in many physiological processes [50, 51], its role in NP and effects upon RIP3 have not been described. Here, we provide the first evidence for a link between ITCH and RIP3 in astrocytes. Knockdown of ITCH decreased the ubiquitination of RIP3 and furthermore promoted the upregulation of RIP3 by mTOR overexpression. These results suggest that ITCH acts as an E3 ligase involved in the regulation of RIP3 ubiquitination by mTOR, although the exact mechanisms underlying this relationship remain to be elucidated. Two major pathways that regulate protein degradation and interact with each other include the autophagy lysosomal pathway and UPS [31, 52]. RIP3, as ubiquitinated by the E3 ligase CHIP, has been reported to be degraded by lysosomes [53]. Here, our results suggest that a selective autophagy may represent a novel mechanism involved with mediating RIP3 degradation. Amino acid starvation induces autophagy to promote RIP3 degradation, and conversely, RIP3 accumulation occurs upon inhibition of autophagy by an overexpression of mTOR. ITCH-mediated RIP3 degradation can be disrupted by CQ, but is not affected by MG132. Such findings, indicate that ITCH specifically regulates RIP3 degradation through autophagy. Thus, the results of our study suggest that the mTOR/ITCH axis regulates ubiquitination degradation of RIP3 through the autophagy pathway.

The management of NP has always faced significant challenges due to the limited number of drug options and poor efficacy of drugs that are available. Most existing treatment methods have been developed as based on observations from clinical experience, rather than targeting specific underlying mechanisms [54]. The lack of an understanding regarding the pathophysiological mechanisms of NP represents the biggest obstacle in developing targeted therapies [2]. Recently, the contribution of glial cells has attracted considerable attention and has become an important area of investigation in NP research [55]. However, due to the lack of specificity of research tools, it is not possible to draw strong inferences from glial cell inhibitors as related to the alleviation of NP [56, 57]. Here, we utilized specific virus-containing cell promoters to achieve a specific gene delivery to astrocytes. In this way, it was possible to identify the precise factors responsible for astrocyte activation and neuronal effects. As a result, important new insights into this field can be achieved, which enhances the translational potential for our research. Unlike existing treatment measures that work through general pain regulation and/or an inhibition of neuronal activity [58], by targeting the mTOR-RIP3 pathway, as based on our current results, there exists the potential for development of highly effective and safe personalized treatment protocols. In this way, it will be possible to surmount the problems of low efficacy, adverse side effects and insufficient relief for patients at tolerated doses, as associated with current treatments [59]. An important consideration in our study that will require further investigation is that no female animal models were included. It is well established that significant differences in molecular, cellular, and systemic mechanisms of pain exist between male and female rodents as well as in humans [60]. Therefore, as part of our future investigations, we plan to examine these effects within female animal models to determine whether similar effects/mechanisms may be present.

To summarize, our study demonstrates that mTOR can serve as a crucial signaling molecule involved in the activation of A1 astrocytes, neuroinflammation and central sensitization in a CCI-induced rat model of NP. We also provide compelling evidence that RIP3 represents a mandatory link enabling mTOR to trigger a cascade of events eventually leading to NP. Therefore, based on these findings, we suggest that mTOR and RIP3 can be considered as potential targets for the treatment of NP.

## Supplementary Information

Below is the link to the electronic supplementary material.

Supplementary file1 (DOCX 1139 KB)

## Data Availability

No datasets were generated or analysed during the current study.
