# Supplementary material for: MTOR Promotes Astrocyte Activation and Participates in Neuropathic Pain through an Upregulation of RIP3
Source: Neurochem Res. 2025 Feb 1;50(2):93. doi: 10.1007/s11064-025-04341-x (PMC11787194; doi:10.1007/s11064-025-04341-x)
Supplement: Supplementary file 1 — Supplementary file1 (DOCX 1139 KB) [file 11064_2025_4341_MOESM1_ESM.docx]

Supplemental Table 1. Key resource table

| **REAGENT or RESOURCE** | **SOURCE** | **IDENTIFIER** |
| --- | --- | --- |
| Antibodies |  |  |
| Rabbit anti-mTOR | Cell Signaling Technology | #2983; RRID:AB_2105622 |
| Rabbit anti-p-mTOR (Ser2448) | Cell Signaling Technology | #5536; RRID:AB_10691552 |
| Rabbit anti-GFAP | Proteintech | #16825-1-AP; RRID:AB_2109646 |
| Rabbit anti-GFP | Proteintech | #50430-2-AP;  RRID: AB_11042881 |
| Rabbit anti-RIP3 | Abcam | #ab62344; RRID:AB_956268 |
| Rabbit anti-p-JNK | Huabio | #ET1609-42;  RRID:AB_3069852 |
| Rabbit anti-GAPDH | Good Here | #AB-P-R001; RRID:AB_3096355 |
| Mouse anti-GFAP | Cell Signaling Technology | #3670; RRID:AB_561049 |
| Mouse anti-IBA1 | Abcam | #ab283319; RRID:AB_2924797 |
| Mouse anti-NEUN | Proteintech | #66836-1-lg; RRID:AB_2882179 |
| Goat anti-C3d | R&D | #AF2655; RRID:AB_2066622 |
| Rabbit anti-TNF-α | ABclonal | #A11534; RRID:AB_2758597 |
| Rabbit anti-c-Fos | Cell Signaling Technology | #2250; RRID:AB_2247211 |
| Rabbit anti-GS | Proteintech | #11037-2-AP; RRID:AB_2110650 |
| Rabbit anti-TSC2 | Cell Signaling Technology | #4308; RRID:AB_10547134 |
| Mouse anti-Ub | Cell Signaling Technology | #3936; RRID:AB_331292 |
| Rabbit anti-XIAP | Cell Signaling Technology | #2042; RRID:AB_2214870 |
| Rabbit anti-PML | Abcam | #ab179466; RRID:AB_2891128 |
| Rabbit anti-RAG1 | Abcam | #ab172637; RRID: AB_3101878 |
| Rabbit anti-ITCH | Cell Signaling Technology | #12117; RRID:AB_2797822 |
| Mouse anti-ITCH | Proteintech | #67757-1-lg; RRID:AB_2918525 |
| Rabbit anti-p62 | Cell Signaling Technology | #8025; RRID:AB_10859911 |
| Rabbit anti-FLAG | Proteintech | #20543-1-AP; RRID:AB_11232216 |
| Rabbit anti-HA | Proteintech | #51064-2-AP; RRID:AB_11042321 |
| Chemicals, peptides, and recombinant proteins |  |  |
| GSK872 | Abcam | #ab254395 |
| Rapamycin | MCE | #HY-10219 |
| Chloroquine | MCE | #HY-17589A |
| SAR405 | MCE | #HY-12481 |
| MG132 | MCE | #HY-13259 |
| EBSS | Solarbio | #H2020 |
| DAB | ZSGBBIO technology | #ZLI-9018 |
| DMSO | MCE | #HY-Y0320 |
| DAPI | Abcam | #ab104139 |
| Lipofectamine 3000 | Invitrogen | #3000015 |
| RIPA lysis buffer | Beyotime | #P0013C |
| Protein A/G agarose beads | Santa Cruz | #sc-2003 |
| Puromycin | Solarbio | #P8230 |

Supplemental Table 2. Primers for RT-PCR

| Target genes | Forward (5’ - 3’) | Reverse (5’ - 3’) |
| --- | --- | --- |
| IL-6 | TTTCTCTCCGCAAGAGACTTCC | TGTGGGTGGTATCCTCTGTGA |
| IL-1β | GGGATGATGACGACCTGCTA | ACAGCACGAGGCATTTTTGT |
| GAPDH | TCTCTGCTCCTCCCTGTTCT | ATCCGTTCACACCGACCTTC |
| TSC2 | GCCATGTGGTTCATTAGGTGC | GGTGCTCCGTGCTCTGAACT |
| RIP3 | TCGTGGGCTCTGAAGAACTG | ACCATAGCCTTCACCTCCCT |
| S100A10 | CATCCCAAATGGAGCATGCCA | GGAACTCCCTTTCCATGAGCA |
| C3D | AGTGTAAGCCGGTAGGACCA | TGGTGGGCAGGTGATTTCTTT |


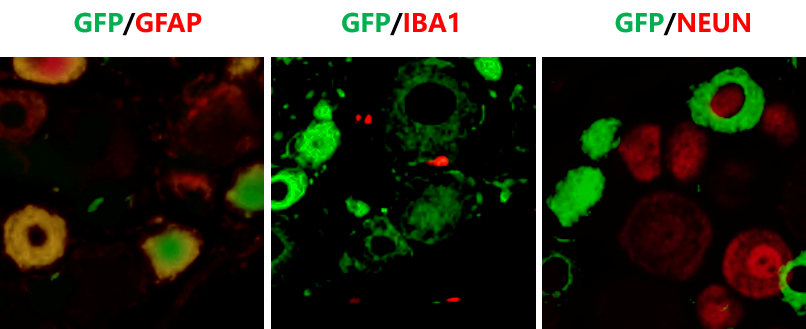


Supplementary Fig. S1. The colocalization immunofluorescence of GFP (green staining) with glial fibrillary acidic protein (GFAP, a satellite cell marker, red staining), ionized calcium-binding adapter molecule 1 (Iba1, a macrophages marker, red staining), and neuron-specific nuclear protein (NEUN, a neuronal marker, red staining) in the dorsal root ganglion.


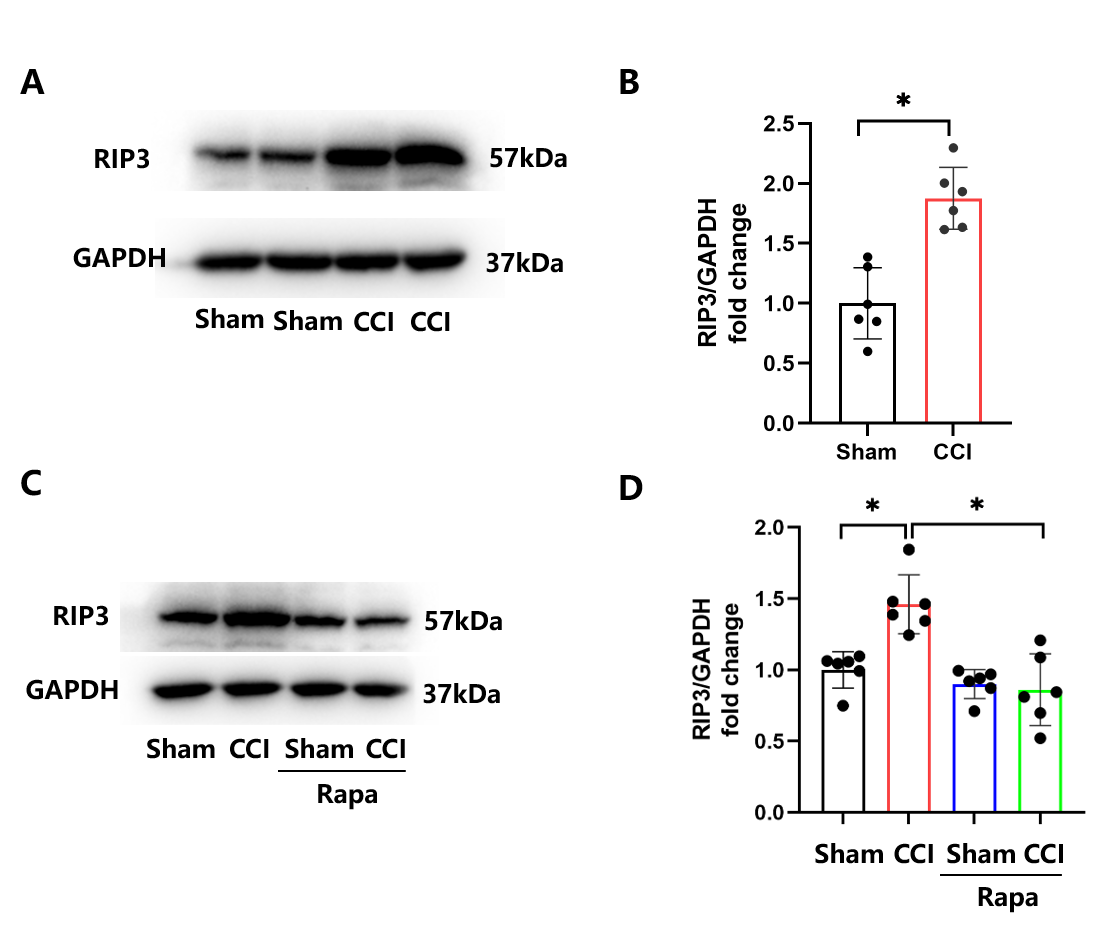


Supplementary Fig. S2. (A, B) Western blotting and quantification of the RIP3 to GAPDH ratio in Sham and CCI rats. (C, D) Western blotting and quantification of the RIP3 to GAPDH ratio in CCI rats treated with the mTOR inhibitor, rapamycin. F= 13.79. (*p < 0.05, n = 6 in each group)


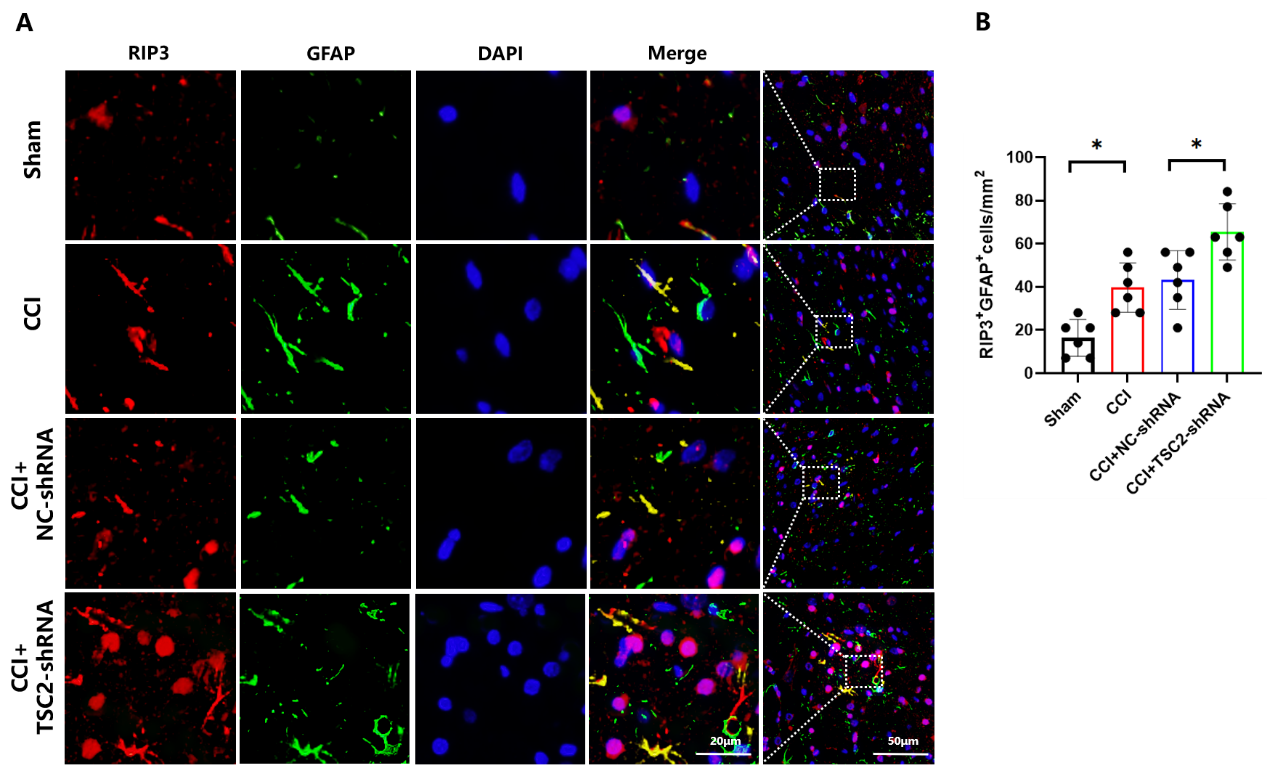


Supplementary Fig. S3. (A, B) Double-immunofluorescence of RIP3 (red staining) and GFAP (green staining) in sham and CCI rats treated with NC-shRNA or TSC2-shRNA.F= 17.34. (*p < 0.05, n = 6 in each group)


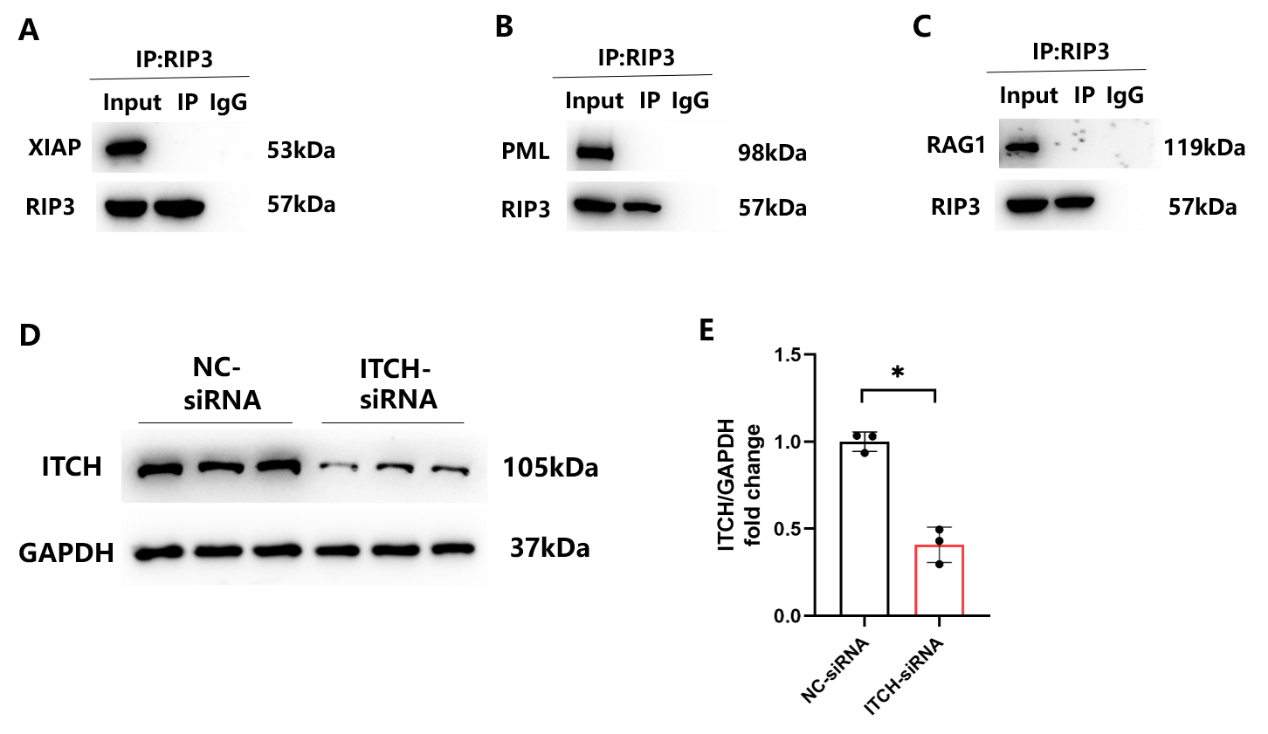


Supplementary Fig. S4. (A-C) Prepare cell lysates and immunoprecipitate with normal IgG or anti-RIP3 antibodies. Co-IP illustrated an absence of any interaction of XIAP(A) /PML(B) /RAG1(C) with RIP3. (D, E) Protein expression of ITCH in NC-siRNA and ITCH-siRNA astrocytes as determined and quantified using western blots. (*p < 0.05, n = 3 in each group)


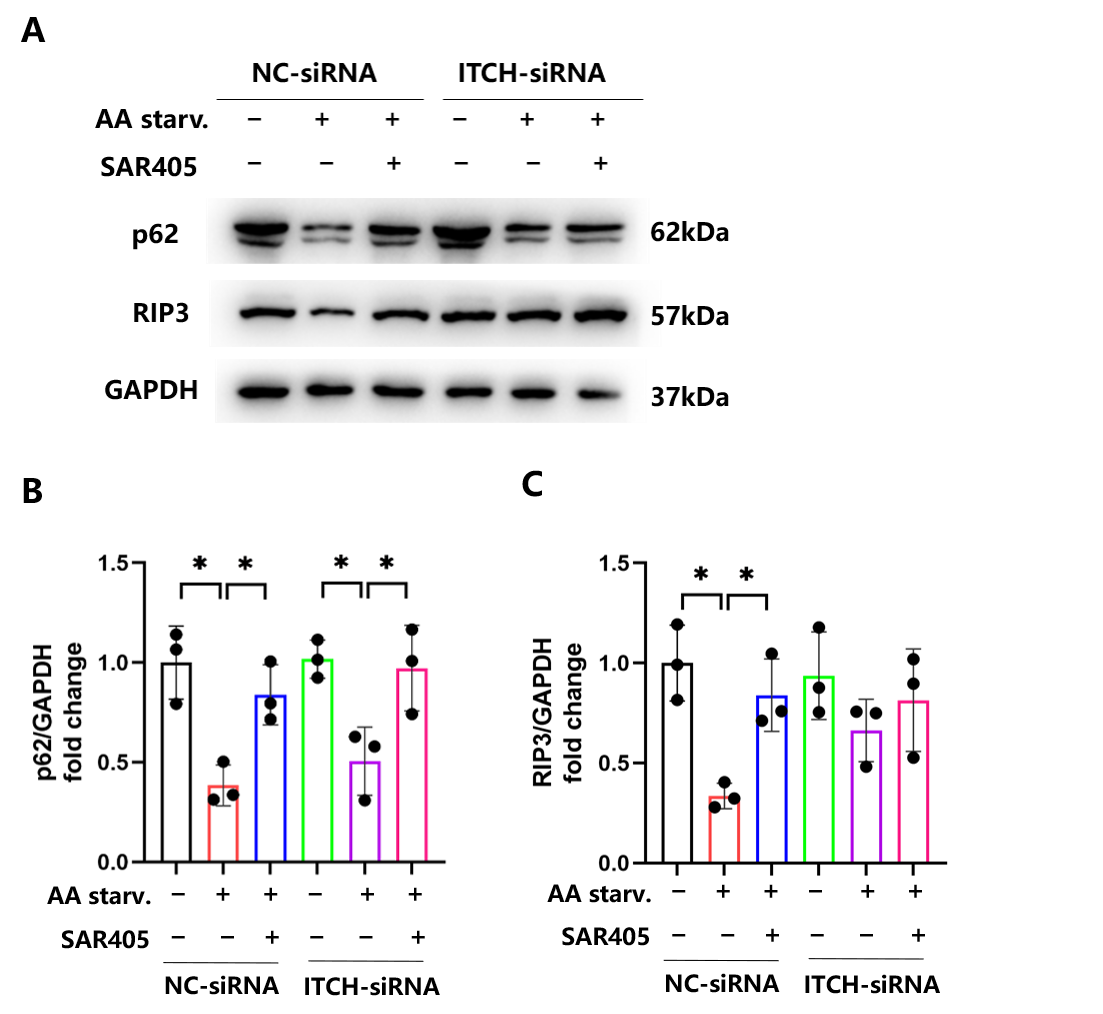


Supplementary Fig. S5. (A-C) NC-siRNA and ITCH-siRNA astrocytes were treated with EBSS medium with or without 10μM SAR405 for 24 hours. Protein expressions of p62 and RIP3 were determined and quantified using western blots. F(B)=8.991, F(C)= 4.907. (*p < 0.05, n = 3 in each group)
